# A Mini Review: Recent Advances in Surface Modification of Porous Silicon

**DOI:** 10.3390/ma11122557

**Published:** 2018-12-15

**Authors:** Seo Hyeon Lee, Jae Seung Kang, Dokyoung Kim

**Affiliations:** 1 Department of Biomedical Science, Graduate School, Kyung Hee University, Seoul 02447, Korea; lee19911230@gmail.com; 2 Laboratory of Vitamin C and Anti-Oxidant Immunology, Department of Anatomy and Cell Biology, College of Medicine, Seoul National University, Seoul 03080, Korea; 3 Institute of Allergy and Clinical Immunology, Medical Research Center, Seoul National University, Seoul 03080, Korea; 4 Department of Anatomy and Neurobiology, College of Medicine, Kyung Hee University, Seoul 02447, Korea; 5 Center for Converging Humanities, Kyung Hee University, Seoul 02447, Korea; 6 Biomedical Science Institute, Kyung Hee University, Seoul 02447, Korea

**Keywords:** surface modification, porous silicon, silicon surface, carbonization, oxidation

## Abstract

Porous silicon has been utilized within a wide spectrum of industries, as well as being used in basic research for engineering and biomedical fields. Recently, surface modification methods have been constantly coming under the spotlight, mostly in regard to maximizing its purpose of use. Within this review, we will introduce porous silicon, the experimentation preparatory methods, the properties of the surface of porous silicon, and both more conventional as well as newly developed surface modification methods that have assisted in attempting to overcome the many drawbacks we see in the existing methods. The main aim of this review is to highlight and give useful insight into improving the properties of porous silicon, and create a focused description of the surface modification methods.

## 1. Introduction

Porous silicon (abbreviated as pSi) is a silicon formulation that has introduced nanopores in its microstructure. Porous silicon was discovered in the mid-1950s, and its unique physical, chemical, optical, and biological properties allowed us to develop new disciplines [1].

**Figure 1:**
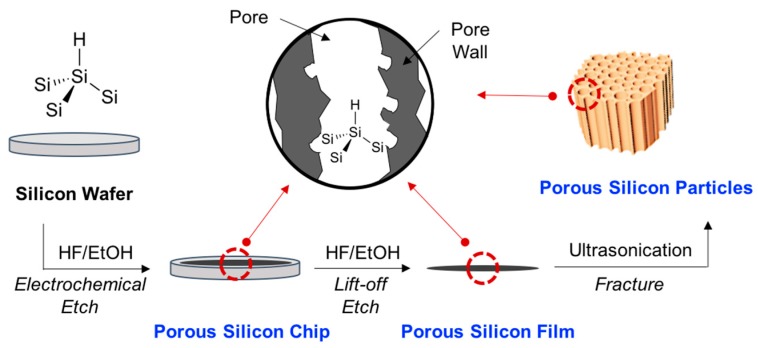
Schematic illustration for the preparation of porous silicon by electrochemical etching and ultrasonication. A porous silicon layer is generated on the surface of the silicon wafer using electro chemical etching, and the layer can be separated from the wafer using lift-off etch. Porous silicon micro- and nano-particles can be prepared using ultrasonication fracturing. The surface of the resulting porous silicon is covered mainly with silicon-hydrogen (Si–H) and partly with silicon-oxygen (Si–OH, Si–O–Si).

Porous silicon can be generated by electrochemical etching of crystalline silicon in hydrofluoric acid (HF) containing aqueous or non-aqueous electrolytes [2]. Silicon (Si) elements in Si wafer can be dissolved out to a hexafluorosilane (SiF_6_^2−^) ion in the electrochemical etching stage, with each wafer generating a different pore diameter; p-type Si wafer (micropores, <2 nm), p^+^/p^++^/n^+^-type Si wafer (mesopores, 2–50 nm), and n^+^-type Si wafer (macropores, >50 nm) [1]. So far, various types of pSi materials have been reported, including pSi chip, pSi film, and pSi micro- and nano-particles (Figure 1). The porosity, pore size, pore pattern, and particle size can be controlled by the fabrication parameters; HF concentration, current density, electrolyte composition, and wafer (dopant type, dopant density, crystallographic orientation) [3]. As compared to anodization and sonication processes, recently a new concept in electroless etching of Si powder has been reported that is an easily scalable process for the generation of pSi particles [4].

The pSi materials have been widely used in various industries and basic science. Due to a significant amount of research and the discovery of quantum confinement effects, photoluminescence, and photonic crystal properties of pSi [5,6,7,8,9], the focus has mostly been on creating optoelectronic materials [10,11], displays [12,13], sensors [14,15,16], and bio-imaging materials [17,18]. Recently, the pSi micro- and nano-particles have been applied to drug delivery systems and controlled release systems, by using the biodegradation property of pSi [18,19,20,21,22]. One such approach, “drug loading in the pore and surface functionalization of pSi materials with disease targeting moiety”, was one of the biggest leaps in the field of drug delivery systems. In addition, the nanostructured pSi material is a promising anode material for high-performance lithium-ion batteries [23,24]. With proper surface modification, pSi materials have shown the suppression of pulverization, low volume expansion, and a long-term cycling stability in the lithiation and delithiation stages as next-generation lithium-ion batteries.

As we described above, the surface modification of pSi materials is imperative to improve the properties of pSi and its usage. Essentially, freshly etched pSi has silicon hydrides (Si–H) on the surface and residual oxides or fluorides are removed by the HF electrolyte (Figure 1). The reactive silicon hydrides on the large surface of pSi is susceptible to slow oxidation in humid air [25,26]. In the field of optoelectronics or battery application, the oxidized pSi could degrade performance of materials. However, the oxidized pSi is necessary in the development of sensors, photoluminescent bio-imaging materials, and drug-delivery systems. Accordingly, surface modification is the most important component in terms of the use of pSi materials.

In this review, we have summarized the conventional surface modification methods, newly reported surface modification methods, and our perspectives.

## 2. Conventional Surface Modification Methods

We categorized the well-known and typical surface modification methods into three categories: (i) hydrosilylation & carbonization, (ii) oxidation, and (iii) hydrolytic condensation.

### 2.1. Hydrosilylation & Carbonization

**Figure 2:**
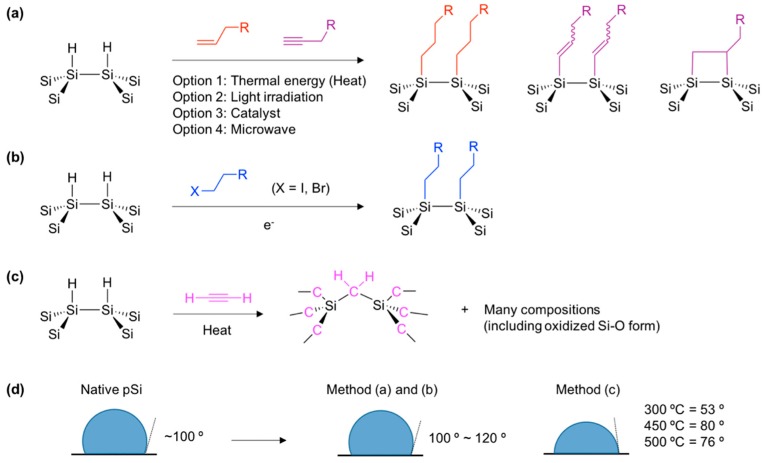
Surface chemistry for native porous silicon (pSi, Si–H). (**a**) Hydrosilylation with unsaturated carbon containing reagents. (**b**) Electrochemical grafting with halide containing reagents. (**c**) Carbonization with acetylene. (**d**) Water contact angle of native pSi and its surface chemistry product. R = simple aliphatic chain, functional group containing aliphatic chain.

Over the last several decades, various attempts have been made at stabilizing the surface of porous silicon in order to improve its suitability for various applications [27,28,29,30,31]. Among them, the silicon-carbon (Si–C) bond formation yielded a very stable surface of pSi due to the low electronegativity of carbon, which possesses greater kinetic stability in comparison to silicon-oxygen (Si–O) [32]. The most ubiquitous reaction to make the Si–C bond from hydrogen-terminated pSi (Si–H) is hydrosilylation. Silicon hydride (Si–H) moiety can react with unsaturated carbon (alkene or alkyne) and bonds to one end of the hydrocarbon reagent (Figure 2) [33].

In the reported methods, the hydrosilylation reaction is achieved through thermal (heat), photon (light), catalyst, and microwave energy (Figure 2a). The thermal and light-induced hydrosilylation provides a means to place a wide variety of functional groups on a pSi surface that allow further surface functionalization, including carboxylic acid. The first hydrosilylation methods were demonstrated by Buriak and co-workers in early 1999, and elaborated by Boukherroub, Chazalviel, Lockwood, and many others afterwards [1,32,33,34,35].

At a similar time, Sailor and Salonen reported electrochemical reduction of organohalides and thermally-induced carbonization approaches using acetylene for the surface grafting of native pSi surfaces (Si–H) (Figure 2b,c) [36,37,38]. The electrochemical reduction method allows surface grafting with a methyl group, which is impossible to achieve through typical thermally induced hydrosilylation. The reaction of pSi with gas phase acetylene generates many derivatives of Si–C bonds on the surface of pSi that is stable in aqueous media. In the water contact angle measurement, typical hydrosilylation with simple aliphatic chains and an electrochemical reaction with methylhalide gives a contact angle of around 100–120° and a hydrophobic surface, but the carbonization reaction gives lower contact angle values at around 53–80°, which might vary depending on the reaction temperature, due to the undesired Si–O bond formation (Figure 2d).

The important points of hydrosilylation and carbonization reactions on the native silicon hydride terminal of the pSi surface can be summarized as: (i) maintaining porous structure, (ii) enhancing the stability of the pSi surface, (iii) making further functionalization possible, and (iv) expanding the scope of application.

### 2.2. Oxidation

The oxide-layer formation on the surface of pSi is also important to functionalize the materials. The hydrophobic property of native pSi became hydrophilic after the surface oxidation. This formulation can be used for bio-related applications such as biosensors, drug delivery systems, and photoluminescence bio-imaging. Generally, silicon-hydride (Si–H) and silicon-silicon (Si–Si) bonds on the surface of silicon materials can be broken with the presence of an oxidant and generates the oxide layer; hydrated silicon oxide (Si–OH) and silicon oxide (Si–O–Si) [39].

**Figure 3:**
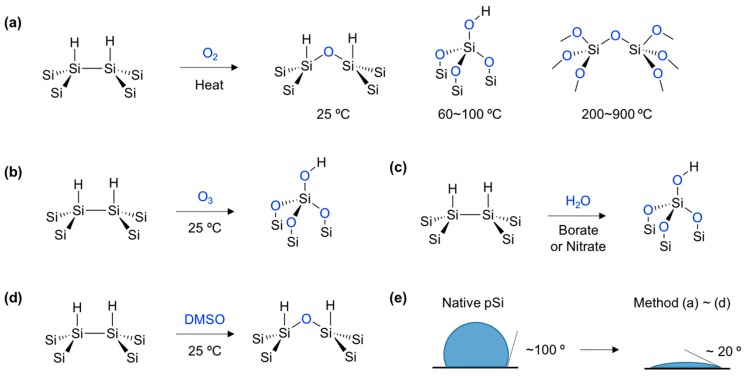
Surface oxidation of native porous silicon (pSi, Si–H). (**a**) Oxidation with a gas-phase oxidant, O_2_, in different temperatures. (**b**) Ozone (O_3_) oxidation. (**c**) Oxidation in aqueous media (H_2_O) with an oxidant (borate or nitrate). (**d**) Oxidation by organic oxidant (DMSO). (**e**) Water contact angle of native pSi and its surface oxidized product.

As an easy and facile method, gas-phase oxidants such as air (including oxygen) and ozone (O_3_) have been widely used for the oxidation of pSi (Figure 3a,b) [25,40]. At room temperature (25 °C), a fairly thin oxide layer (Si–O–Si) grows over the course of several months in the presence of oxygen. At temperatures below 200 °C, pSi generates more hydroxy species silicon oxide layers on the surface. Oxidation at 900 °C is sufficient to completely convert the pSi to silicon oxide, which has no crystallinity of silicon, although the transformation depends on the type of pSi. Interestingly, ozone oxidation at a room temperature of 25 °C gives a more hydrated silicon oxide than the thermal oxidation procedure below 200 °C. 

Solution based oxidation of pSi is also widely used due to the facile operation and controllable oxidation states. In aqueous solutions, a silicon atom on the pSi surface can make a 5-coordinate intermediate with water molecules (H_2_O) and its cascade condensation, giving a Si–O–Si and hydroxylated Si–OH oxide layer (Figure 3c) [41]. The oxidation rate and thickness of the oxide layer also can be boosted or regulated in the presence of an additional oxidant, such as borate [42] or nitrate [43]. Similarly, organic solvents can also be used. Dimethyl sulfoxide (DMSO) can also oxidize the surface of pSi in a mild way and generate the Si–O–Si layer on the surface [44]. The Deuterium tracer study revealed that DMSO tended to break the Si–Si rather than Si–H bond (Figure 3d).

In terms of bonding energy, the Si–Si bond is weaker than the Si–H bond, so the mild oxidant (DMSO, pyridine, etc.) [45] and thermal condition (25 °C) tends to break the Si–Si bond, and generate a Si–O–Si layer on the surface. Conversely, relatively reactive oxidants (O_3_, borate, nitrate, etc.) and harsh conditions (>100 °C) tend to break both the Si–Si and Si–H and generate Si–O–Si and Si–OH. The generation of an oxide layer on the pSi surface can be monitored by the water contact angle measurement, and all oxidation products show hydrophilic properties below a 20° angle (Figure 3e).

The important point to consider about the oxidation of the native pSi surface can be summarized as: (i) enhancing hydrophilicity utilizing its bio-related work, (ii) improving functionalization as much as possible, (iii) enhancing quantum confinement effects on the surface, and (iv) enhancing the generation of the reactive Si–O intermediate to improve modification. 

### 2.3. Hydrolytic Condensation

**Figure 4:**
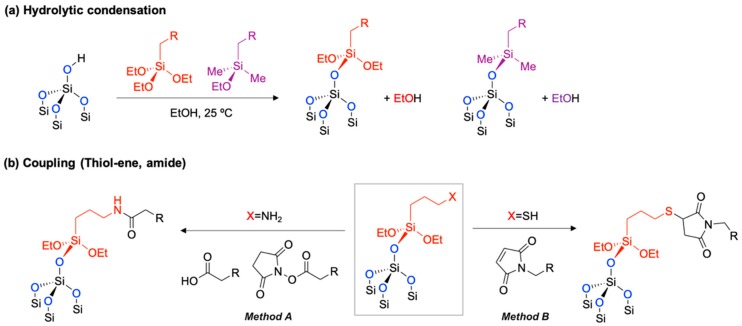
Surface modification of oxidized pSi. (**a**) Hydrolytic condensation with silanol reagents. (**b**) Further functionalization of hydrolytic condensation products via amide coupling (Method A) and thiol-ene addition (Method B). A box in Figure (**b**) indicates the surface functionality of hydrolytic condensation with APTES or MPTES on the oxidized pSi.

The oxidized pSi is a good platform for further surface modification. The hydrolytic condensation with organotrialkoxysilane reagents generates new Si–O–Si bonds on the surface in a protic solvent, such as ethanol at mild temperatures (25 °C) (Figure 4a) [46]. This simple silanol chemistry is well-known and widely used within bio-related works, such as biomolecule conjugation, PEGylation (polyethylene glycol attachment reaction), and controlled degradation of pSi materials in bio-fluid [17,18,47,48,49]. In tests, there are two standard coupling reagents; (i) 3-aminopropyltriethoxy-silane (APTES, X = NH_2_ in Figure 4b) [50,51], and (ii) 3-mercaptopropyltriethoxy-silane (MPTES, X = SH in Figure 4b). The hydrolytic condensation product with APTES gives primary amine terminals on the surface of pSi, and this amine moiety can be chemically conjugated using proteins, DNA, antibodies, drugs, and many other molecules, via amide coupling with carboxylic acid or *N*-hydroxysuccinimide esters (Method A) [52,53]. In a similar way, the hydrolytic condensation product with MPTES gives a thiol terminal on the surface of pSi, and it can be conjugated with substrates via a thiol-ene reaction (also known as alkene hydrothiolation) with maleimide (Method B) [54]. Both chemistries show sufficiently high yields of reaction for most of the oxidized pSi materials. Recently, further functionalization of oxidized pSi using metal-free bioorthogonal click chemistry was applied for the peptide conjugation [55,56].

## 3. Recently Developed Surface Modification Methods

In the previous chapter, we summarized the existing surface modification methods for pSi materials. Although the known methods show superior surface modification abilities, the practical applications in industry and basic sciences have met difficulties for many reasons. 

As an example, the thermally induced hydrosilylation of native pSi typically required high heat (>200 °C), a very specific light with sufficiently high power, an air-sensitive catalyst, such as rhodium complex (known as Wilkinson’s catalyst), and special instruments. This kind of experiment could be conducted by an expert in an inert atmosphere like a nitrogen glovebox with home-built instruments. Occasionally, the resulting pSi material showed low efficiency of surface conversion and non-uniformed morphology. Surface modification by oxidation and hydrolytic condensation also came with problems, such as a loss of pSi during modification, long reaction times (> 12 h), undesired cross-linking of pSi, and low efficiency because of generation of the by-product. To overcome these issues, new surface modification methods have been developed recently, such as (i) thermally induced dehydrocoupling, (ii) ring-opening click chemistry, and (iii) calcium- or magnesium-silicate formation. This chapter has summarized the key points of these recent advances in surface modification of pSi materials. 

### 3.1. Thermally Induced Dehydrocoupling

**Figure 5:**
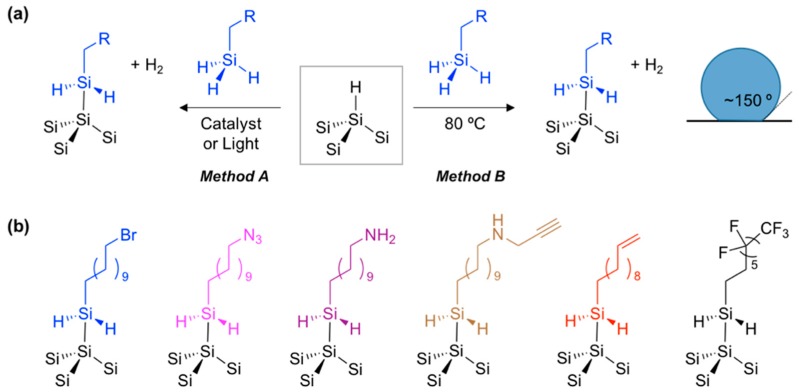
Thermally induced dehydrocoupling of the native pSi surface (Si–H) with trihydridosilane reagents (H_3_Si-CH_2_-R, marked in blue). (**a**) Dehydrocoupling by catalyst or light (Method A) and mild heat (Method B). Inset figure: water contact angle of reaction product. (**b**) Summary of reaction products of pSi with various trihydridosilane reagents.

**Figure 6:**
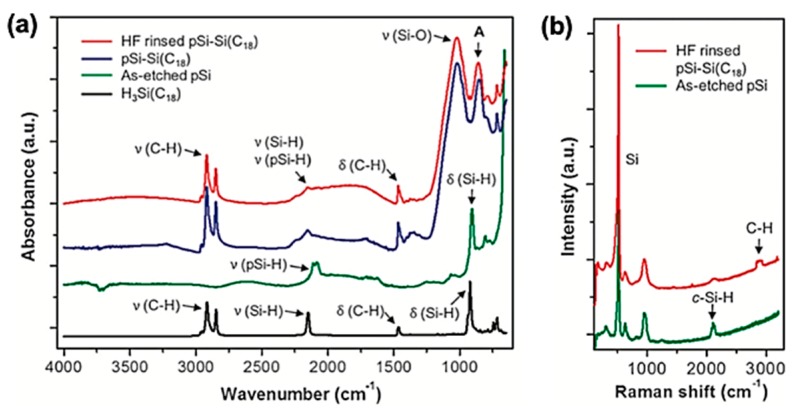

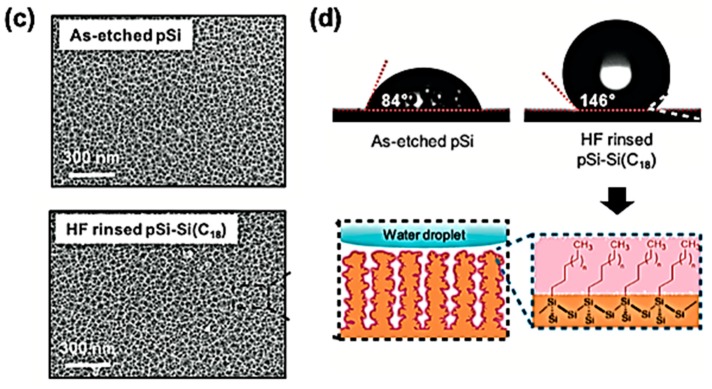
Characterization of a thermally induced dehydrocoupling reaction product of pSi with octadecysilane (H_3_Si-C_18_). (**a**) Attenuated total reflectance Fourier-transform infrared (ATR-FTIR) spectra of the reagent (H_3_Si-C_18_) and products (pSi-Si-C_18_). (**b**) Raman spectra obtained before (As-etched pSi) and after reaction (HF rinsed pSi-Si-C_18_). (**c**) Scanning electron microscope (SEM) images of pSi before (As-etched pSi) and after reaction (HF rinsed pSi-Si-C_18_) (plan view). (**d**) Water contact angle image and illustration of the surface after reaction. Reproduced with permission from [58]. Copyright 2016 Wiley-VCH.

The hydrosilylation reaction of the native pSi material, with alkene of alkyne moieties on the organic reagent, requires an exclusion of oxidants including air, moisture, and water in order to avoid production of an undesired silicon oxide. An alternative method is dehydrogenative coupling with a trihydridosilane reagent (H_3_Si-R, R = aliphatic, aromatic) (Method A, Figure 5a) [57]. However, this dehydrogenative coupling method requires transition metal catalysts in order to effect the transformation, and the highly reactive catalysts can lead to an oxidative side reaction. Very recently, Kim and co-workers reported the non-catalytic dehydrocoupling reaction can proceed under mild thermal conditions on the pSi surface (Method B, Figure 5b) [58,59]. The first investigation was successfully carried out with the pSi film and an octadecysilane, H_3_Si(CH_2_)_17_CH_3_ (abbreviated as H_3_Si-C_18_). The pSi film was heated at 80 °C for 1–24 hours, in the presence of a neat silane reagent. Infrared spectrum analysis of native the pSi film and reaction product (pSi-Si(C_18_)) displayed bands associated with pSi-H and an organosilane reagent (Figure 6a). A strong Si lattice mode at 515 cm^−1^ was observed in the Raman spectrum of pSi-H and pSi-Si(C_18_), which indicated a retained crystallinity of pSi after the reaction (Figure 6b). The preservation of an open pore structure was observed by scanning electron microscope (SEM) images (Figure 6c). The superhydrophobic surface property was observed in the water contact angle measurement (>150°) after rinsing with HF (Figure 6d), and it suggests that this reaction occurred between the Si element on the pSi and the Si element on the reagent, not through the oxidized pSi surface.

The thermally induced dehydrocoupling reaction with a trihydridosilane reagent shows remarkable tolerance to oxidants such as air and water. The reaction of pSi with a mixture of water and octadecysilane in open air conditions shows an adverse effect, and a grafting reaction. They also followed the effect of surface modification on the intrinsic photoluminescence quantum-confined pSi material. For this study, n-type pSi silicon film was prepared by electrochemical etching under UV light irradiation. Interestingly, the preserved photoluminescence was monitored after the reaction, and the product maintained their emissions for a long time (> 9 days) within the aerated water whilst immersed. They also demonstrated dehydrocoupling reactions with various trihydridosilane reagents and tested their suitability for further organic functionalization reaction. Many functional groups such as bromide, azide, primary amine, propargyl, and perfluorocarbon were able to be introduced onto the surface of the resulting productions. Further conjugation chemistry and electrostatic drug loading demonstrations were successfully carried out with a primary amine-terminal production toward fluorescein isothiocyanate and negatively charged drugs (ciprofloxacin in this study). 

### 3.2. Ring-opening Click Chemistry

Driven by the desire for functionalization and stabilization of the pSi surface, the reactive Si-H surface often converted to Si–O moiety. As we described in the previous chapter, oxidized pSi surfaces can be functionalized by the grafting of organotrialkoxysilanes via a hydrolytic condensation reaction. Despite their utility, the critical limitations, such as undesired cross-linking, which results in an overly thick coating or clogging of the pore of pSi, long reaction times, and low coupling efficiency give many drawbacks to the practical applications of pSi [1,60].

**Figure 7:**
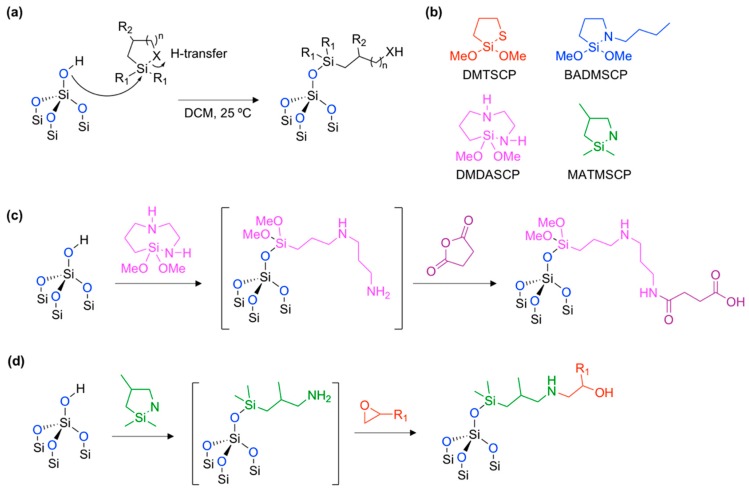
Ring-opening click chemistry of oxidized pSi surface (Si–OH) with cyclic-silane reagent. (**a**) Reaction mechanism. DCM: dicholoromethane. R_1_ = OMe, Me. R_2_ = H, Me. X = S, N. (**b**) Cyclic-silane reagents. (**c**,**d**) Further functionalization of ring-opening click chemistry products (pink and olive) via amide coupling with succinic anhydride (purple) and tandem coupling with epoxy containing reagent (red). DMTSCP: 2,2-dimethoxy-1-thia-2-silacyclopentane. BADMSCP: *N*-*n*-butyl-aza-2,2-dimethoxy-silacyclopentane. DMDASCP: 2,2-dimethoxy-1,6-diaza-2-silacyclooctane. MATMSCP: N-methyl-aza-2,2,4-trimethyl-silacyclopentane.

In 2016, Kim and co-workers developed a facile surface modification method of oxidized pSi via ring-opening click chemistry, using 5-membered heterocyclic compounds containing Si–S or Si–N bonds within the ring (Figure 7) [61]. 

The name of the reaction originated from simple click chemistry that showed a lack of by-product with a high yield and wide scope of applicability. The hydroxy moiety on the oxidized pSi surface attacked the silicon element on the cyclic-silane reagent and the Si–X (X = S or N) bond was broken (ring-opening) with a proton transfer (H-transfer) within a mild condition (Figure 7a). Originally, this kind of click chemistry was proposed by Arkles and co-workers in Gelest Inc. USA in 2015 [62]. They found an unusually fast and efficient surface grafting ability of the cyclic azasilane reagent (Si–N bond in the ring). With promising results based on the amorphous silica materials, Kim and co-workers expanded the research field to oxidized pSi films and pSi micro- and nano-particles using other heterocyclic silanes (Figure 7b). The thermogravimetric analysis (TGA) of the resulting production, prepared through hydrolytic condensation and ring-opening click chemistry, gave ~2–4% and ~8% mass changes, representing a high efficiency for this new type of method for surface grafting. The maintenance of the crystallinity of the silicon skeleton was then verified using a powder X-ray diffraction (XRD) measurement. 

Within these promising surface grafting results, a one-pot tandem synthesis was demonstrated, using an amine-containing cyclic azasilane reagent (Figure 7c,d) [61,63]. The ring-opening click reaction generated a primary amine at the surface, which then reacted to the succinic anhydride or epoxy containing reagent. Through these tandem coupling reactions, new functional groups, such as carboxylic acid (–COOH) or hydroxyl (–OH) moiety were generated, and, therefore, showed a good case for application using bio-conjugation. 

**Figure 8:**
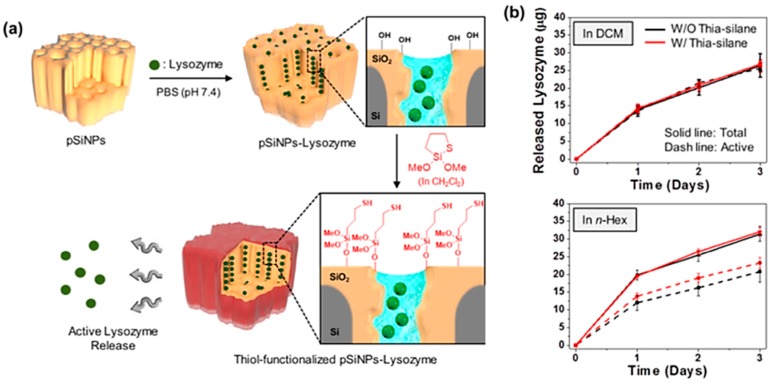
Application of ring-opening click chemistry for substrate loaded pSi nano-particles (pSiNPs). (**a**) procedure used to load lysozyme into pSiNPs and modify the resulting particles with the cyclic-silane reagent (thia-silane in this study). DCM: dichloromethane. (**b**) Release profile of lysozyme from unmodified pSiNPs and thia-silane functionalized pSiNPs that were prepared in different solvents, DCM or *n*-Hex. Reproduced with permission from [61]. Copyright 2016 American Chemical Society.

In this study, the authors also tested the compatibility of the surface functionalization with protein loaded porous silicon nano-particles (pSiNPs) (Figure 8). The nano-particle formulation of pSi maintained its pore structure, its protective sensitive payloads from denaturing in vitro or in vivo, and has proven to be a promising delivery vessel. A model protein lysozyme was loaded into the pSiNPs, and then the surface of resulting pSiNP was functionalized with thia-silane reagent in either DCM or *n*-Hex (Figure 8a). The generation of the surface thiol species was within 72 h and retained its activity; 98% (reaction in DCM) and 72% (reaction in *n*-Hex) (Figure 8b). By contrast, lysozyme loaded pSiNPs with the hydrolytic condensation reaction using MPTES gave only 68% activity of lysozyme after release.

### 3.3. Calcium- or Magnesium-Silicate Formation

**Figure 9:**
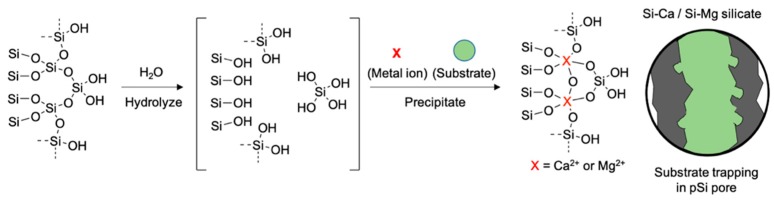
Schematic illustration of the calcium (Ca^2+^)- or magnesium (Mg^2+^)-silicate formation of pSiNPs surface. A thin oxide layer and orthosilicic acid (Si(OH)_4_) is generated from the pSiNPs in aqueous media and forms a precipitate that traps the substrate (olive) within the nanostructure.

A formulation of a pSi core with an oxide layer shell is an attractive platform for further chemical conjugation, offering increased stability, enhanced photoluminescence intensity, and a controlled drug delivery release system. In some cases, the oxide layer formation of pSi has been used to trap the substrates within the nanostructure [43,49,64]. In 2016, Kang and co-workers reported a facile pSi surface oxidation method through the formation of a calcium silicate (Ca-silicate) insoluble shell (Figure 9) [48]. It was shown in this study that the high concentration calcium (II) ion can react with a hydroxylated silicon surface and hydrolyzed orthosilicic acid (Si(OH)_4_) and form the Ca_2_SiO_4_ precipitation (calcium silicate shell) on the surface, trapping the substrate, siRNA. The calcium silicate shell could also be degraded in the aqueous media and release the substrate, but the release rate was slower than the known physical trapping method. In addition, the oxide shell formation process dramatically increased the quantum yield of pSiNPs from 0.1% to 21%. The calcium silicate formation with pore sealing was verified by transmission electron microscope (TEM) images, energy dispersive X-ray (EDC) analysis, Fourier transform infrared (FTIR) spectrum, and nitrogen adsorption–desorption isotherm analysis. 

After basic characterizations, the authors went on to demonstrate a potential use for the disease specific gene delivery approach, by using a modification of the calcium silicate coated pSiNPs (Ca-pSiNPs) surface. First, the surface of the siRNA loaded Ca-pSiNPs (Ca-pSiNP-siRNA, ~20 wt % siRNA) was functionalized with 3-aminopropyl-dimethylethoxysilane (APDMES) via hydrolytic condensation. The Ca-pSiNP-siRNA was then conjugated with a bi-functional polyethyleneglycol (PEG), maleimide-PEG-succinimidyl carboxy methyl ester (MAL-PEG-SCM), via amide coupling between amine and succinimidyl carboxy methyl ester. The remaining distal end of the PEG was additionally conjugated, targeting RVG (rabies virus glycoprotein) and cell penetrating peptides (mTP; myristolated transportan). A mice study was conducted using mice with brain injuries, and it concluded that the formulation was successfully delivered to the injured site and released the active siRNA payload. 

As a follow-up study, Wang and co-workers reported that a similar demonstration based on the magnesium silicate (Mg-silicate) trapping of bovine serum albumin (BSA) into the pSi micro-particles with their photoluminescence intensity analysis [20]. They found that the Mg-silicate can suppress burst releases of the payload, and this kinetic release can be tracked using the photoluminescence of pSi micro-particles. 

In this chapter, we cover more recently developed surface modification methods for pSi materials with their detailed mechanisms, key benefits, and applications. We also summarize the key advantages and disadvantages compared to existing surface modification methods (Table 1). 

## 4. Summary and Outlook

Surface modification can provide physical- or chemical-stability, functionality, and chemically reactive sites, which can be used to interact or conjugate substrates. For this reason, there has been a desperate need for the development of a more advanced surface modification method of the materials. The existing methods have been confronted by many challenges, such as hydrosilylation, oxidation, and hydrolytic condensation. In this focused review, we introduced three recently developed and differing surface modification methods of porous silicon; hydrolytic condensation, ring-opening click chemistry, and calcium- or magnesium-silicate formation. These new methods have shown remarkable advantages compared to more traditional existing methods, in an attempt to develop this research to improve the properties of porous silicon. As scientists undertaking research within the field of material science, especially based on porous silicon, we believe that the next-generation of surface modification methods and reagents for porous silicon will have several focuses: (i) ease-of-use, (ii) high reproducibility, (iii) high bio-applicability (low toxicity), (iv) controllable surface modification ratio, (v) minimizing or maximizing the pore collapse during the modification, and (vi) multi-functional surface modified moiety. Advances in surface modification methods and reagents can expand their application throughout many different fields. In particular, those that are currently attracting attention, such as polymer fabrication, food preservation coating, cell growth regulation, tissue engineering, and antifungal coating for medical devices. In addition, this new fabrication technique is expected to be used in certain applications (i.e., water-repellent coating, photolithography, etc.). We hope this review offers some basic information about porous silicon and surface modification methods, and that it can inspire other scientists to develop more advanced methods in order to improve these materials.

## Figures and Tables

**Table 1:** Summary of advantages, disadvantages, and applications for each surface modification methods of pSi materials.

Methods	Advantages	Disadvantages
Carbonization (Si–H to Si–C)	- Single-step - Enhance hydrophobicity - Enhance stability	- Requires harsh reaction condition - Requires special instruments - Need practiced hands
Oxidation (Si–H to Si–OH, Si–O–Si)	- Facile method - Enhance hydrophilicity - Bio friendly	- Loss of pSi during modification - Difficult to control oxidation state - Undesired pore clogging
Hydrolytic condensation (Si–OH to Si–O–Si–R)	- Facile method - Functional group diversification - Bio friendly	- Loss of pSi during modification - Time consuming process - Undesired cross-linking of pSi
Thermal dehydrocoupling (Si–H to Si–Si–R)	- Single-step, mild condition - Enhanced hydrophobicity - Functional group diversification	- Undesired Si-O formation - Large amount of reagent (neat)
Ring opening click chemistry (Si–OH to Si–O–Si–R)	- High yield - Single-step, facile method - Functional group diversification - Inert to payload	- Reaction only in aprotic solvent - Only for hydroxylated pSi
Ca-/Mg-silicate formation (Si–OH to Si–O–Ca/Mg–O–Si)	- Single-step, facile method - High loading yield - Photoluminescence generation - Further surface modification	- Exothermic reaction - Require further modification
